# Primary health workers’ knowledge and practices relating to neonatal jaundice in Ibadan, Nigeria

**DOI:** 10.4102/phcfm.v9i1.1081

**Published:** 2017-01-30

**Authors:** Adebola E. Orimadegun, Adeola O. Ojebiyi

**Affiliations:** 1 Institute of Child Health, College of Medicine, University of Ibadan, Ibadan, Nigeria

## Abstract

**Background:**

Over half of births and newborn care occur in primary healthcare facilities in Nigeria, but information on activities of personnel working there is scarce.

**Aim:**

To assess the knowledge and practices relating to neonatal jaundice (NNJ) among community health workers (CHWs) and community birth attendants (CBAs) in Nigeria.

**Setting:**

We conducted a cross-sectional survey of all 227 CHWs and 193 registered CBAs in Ibadan, Nigeria.

**Methods:**

Knowledge and practices regarding NNJ were measured using a pretested questionnaire. Knowledge and practices were assessed on a 33-point scale and a 13-point scale, respectively. Scores ≤ 17 and ≤ 9 was regarded as poor knowledge and as wrong practice, respectively.

**Results:**

Many (64.5%) of the respondents could not correctly describe examination for NNJ (CHWs: 49.4%; CBAs: 50.6%). Of the 200 (47.6%) who treated NNJ 3 months prior to the study, 62.5% (CHWs: 66.9% and CBAs: 53.7%) treated NNJ with orthodox drugs. Drugs prescribed included: antibiotics (93.3%), antimalarials (5.3%), multivitamins (28.0%), paracetamol (6.2%) and phenobarbitone (7.1%). Significantly more CHWs than CBAs practiced exposure to sunlight (33.1% versus 16.4%) and administration of glucose water (28.6% versus 14.9%), while 58.0% of all respondents referred cases to secondary health facilities. Overall, 80.2% had poor knowledge (CHWs: 78.9%; CBAs: 81.9%) and 46.4% engaged in wrong practices (CHWs: 57.3%; CBAs: 33.7%). CHWs were more likely to indulge in wrong practices than CBAs (OR = 2.22, 95% CI = 1.03, 4.79).

**Conclusion:**

Primary Health Workers in Ibadan had poor knowledge and engaged in wrong practices about NNJ. The needs to organise regular training programmes were emphasised.

## Introduction

In sub-Saharan Africa, though the annual rate of reduction of under-five mortality was faster than the rest of the world, Africa continues to experience higher child morbidity and mortality rates as well as greater numbers of developmentally deprived children.^1^ The causes of these development challenges are attributable to early life health problems such as neonatal jaundice (NNJ).^2,3^ Of the estimated 4 million neonatal deaths in the world, approximately 45% occurred in the first 28 days of life (neonatal period), mainly from preventable causes such as prematurity and NNJ.^4^ Tackling neonatal morbidity and mortality remains imperative not only because it contributes a high proportion to under-five deaths, the health interventions needed to address the major causes differ from those needed to address other child deaths.

Jaundice may be clinically significant for many reasons. The high levels of unconjugated bilirubin may cause irreversible brain damage, referred to as kernicterus, and bilirubin toxicity (kernicterus) causes nerve deafness, choreoathetoid cerebral palsy and mental retardation.^5^ A previous report from Ibadan showed that NNJ contributed as much as 3.1% – 7.1% of deaths among newborns admitted to a tertiary health facility in Ibadan in the year 1991 to 2000.^6^ Also, studies conducted in Calabar,^7^ Lagos^8^ and Abakalilki^9^ revealed that NNJ accounted for 10% – 35% of all admissions in the neonate intensive care unit. In addition, many studies have demonstrated increased risk of severe hyperbilirubinaemia and its resultant complication (kernicterus) among Nigerian neonates.^10^ Thus, kernicterus is a leading cause of preventable brain damage, physical and mental handicap and early death among infants in Nigeria.^10,11^ Consequently, kernicterus with its attendant medical, social and economic burden on the patient, family and society at large frequently occurs in many developing countries.^12^ Therefore, hyperbilirubinaemia poses serious problems when it is not appropriately treated and many of the complications are permanent and irreversible. For these reasons, the presence of NNJ requires early diagnoses and prompt treatments by experienced healthcare providers.

According to the World Health Organization (WHO), achieving the MDG 4, that is reducing child mortality by two-thirds, will require universal coverage with key effective, affordable interventions including care for newborns.^13^ In countries with high mortality, such as Nigeria, these interventions could reduce the number of deaths by more than half. Current efforts at reducing NNJ and indeed all neonatal illnesses in developing countries are increasingly focused on early detection of serious neonatal illnesses through screening by minimally trained health workers at home or primary care facilities and referring those requiring hospital-level care.^14^ Notably, in Nigeria, over half of births take place at home, and primary health centres and births in these settings have been associated with significant morbidity and mortality.^15^ The competence of home birth attendants and health workers in primary facilities is central to achieving a favourable outcome for management of NNJ. Therefore, it is expedient that health workers in primary health facilities improve their ability to diagnose NNJ and recognise the need for referral in order to improve outcomes.

Knowing that majority of newborns are cared for at home and at the primary healthcare facilities where community health workers (CHWs) are working underscores the need to find out what they know and current practices in order to proffer solutions to identified gaps. At the moment, there is dearth of state-wide data relating to practices of health workers on treatment of NNJ at the primary care level in Nigeria. This information is necessary for the purpose of formulating strategies and policies geared towards effective care in this respect. Often, the obstacle to change in practice may be a lack of knowledge of the benefits of more effective methods, or lack of knowledge of the problems and severity of associated effect the existing practice may be having. To address the United Nations MDG 4 on reducing childhood mortality, there is the need for better understanding of the levels of knowledge and roles played by CHWs who attend to a remarkable number of ill neonates in resource-limited countries like Nigeria. Therefore, this study was designed to assess the knowledge and practices relating to NNJ among CHWs and community birth attendants (CBAs) in Ibadan, Nigeria.

## Research methods and design

### Study design and settings

This research employed a cross-sectional study design. The primary health facilities and community health posts, which include the CBAs’ homes, were the main centres of this study. The study involved a one-time interaction with CHWs and CBAs while at their duty post. The study was carried out in Ibadan, located in the south west of Nigeria. Ibadan is the third largest metropolitan area in Nigeria, by population, after Lagos and Kano, with a population of over 3 million, and the largest metropolitan geographical area. The principal inhabitants of Ibadan city are the Yoruba-speaking people of south-western Nigeria. Healthcare delivery in Ibadan spans the three tiers of government, that is, the federal, the state and the local government, managing the tertiary, secondary and primary health facilities, respectively. These primary health facilities are located within the communities, at readily accessible locations in order to serve as the first ‘point-of-call’ for healthcare services. These facilities open every day and are manned by CHWs; they also have affiliated homes that are manned by trained CBAs. The primary health clinics (PHCs) operate using the standing order of the federal ministry of health while the CBAs are trained locally to take births and refer to the affiliated PHC which is usually the Comprehensive PHC of the local government area (LGA). On an average, most PHCs open during the day while the CBA homes are open to clients 24 hours of the day.

### Study population

In all, there were 167 officially registered primary health facilities covered in this study. The target population for the study included CHWs (community health officers and community health extension workers) at the primary health centres and CBAs in the 11 local government areas that made up Ibadan. At the time of the study, there were a total of 433 eligible health workers in the 11 LGAs selected for this study. However, only those who were not on leave constituted the sampling frame.

### Sampling technique

All CHWs present at work and all available trained CBAs in the CBAs’ home were selected for the study. The investigator first visited the facility, explained to the head of the facility the study requirements, after which all CHWs, health extension workers and CBAs present at the facility at the time of visit were interviewed. Thus, CHWs that were not at their duty post at the time of recruitment and unregistered CBAs were excluded from the study.

Efforts were made to enrol all eligible health workers during the 8-month study period unless consent was declined. There were 433 health workers eligible to participate in the study, but complete responses were obtained from 420 participants.

### Instrument and data collection procedure

The data for the study were collected using an interviewer-administered semi-structured questionnaire. The items in this questionnaire were adapted from the study by Ogunfowora and Daniel.^10^ The questionnaire had three sections which covered questions on socio-demographic characteristics, knowledge and practice regarding NNJ, respectively. The questionnaire received inputs from two experts on perinatal health at the University of Ibadan to improve its content. The questionnaire was pretested among 25 primary health workers to ensure appropriateness and clarity of questions. Observations during pretest exercise and feedback from respondents were used to improve the construct of the questionnaire. The data were collected by the researcher and trained research assistants within a period of August 2013 – March 2014 and within the normal hours of work (8am – 4pm). Each participant was met by an interviewer, and informed consent was obtained. The interview took an average of 20 min – 25 min.

### Data analysis

The data were entered into and analysed using SPSS 18.0 statistical software (SPSS Inc., Chicago, Illinois, USA). The main outcome variables were knowledge score and practice. The knowledge score for each respondent was determined by allotting a score of ‘1’ to correct response and ‘0’ (zero) to wrong response to questions on the definition of NNJ, colour of urine of neonates with NNJ, dangers signs, causes, treatments and perceived adverse events associated with the management of NNJ. Thus, maximum obtainable knowledge score was 33. A knowledge score of ≤ 17 was considered as poor knowledge while > 17 was categorised as good knowledge. Similarly, the practice score for each respondent was determined by allotting a score of ‘1’ to correct response and ‘0’ (zero) to wrong response to questions on ‘what primary health workers would offer for a case of NNJ?’ and whether or not they would refer cases. These gave a total of 13 maximum obtainable knowledge scores. A primary health worker with a practice score of ≤ 9 was regarded as engaging in wrong practice while a score of > 19 was considered correct practice. The association between practices (wrong or correct) and demographic variables were assessed using odds ratio (OR) and 95% confidence intervals. Logistic regression was carried out to identify variables that independently predicated wrong practices among the three (age, workers’ category and education) found to show significant association at bivariate level of analysis. The inferential statistics were considered statistically significant only if *p*-value was less than 0.05.

## Ethical consideration

The protocol for this study was reviewed and approved by the UI/UCH Institutional Review Committee (Approval Number: UI/EC/12/0007). Each respondent’s consent was obtained using a standardised informed consent form after provision of adequate, clear and complete information about what the study entails. Participation in the study was voluntary. Confidentiality was ensured by using a serial number on the information collected rather than a name. Only the researcher knew the identification, and this information was kept.

## Results

Characteristics of study participants: The gender distribution, mean age and duration of practice of the respondents are given in Table 1. The mean age of CHWs (42.1 years ± 7.7 years) was significantly lower than those of CBAs (49.4 years ± 9.5 years), but the mean duration of practice (work experience) was not significantly different (CHWs: 16.4 years ± 8.6 years; CBAs: 15.8 years ± 9.6 years; *p* = 0.526). With respect to educational attainment of respondents, 8.3% were educated up to primary school, 27.4% had secondary education and 61.9% were educated beyond secondary school, while 2.4% had no formal education. Notably, all respondents who had no formal education were CBAs.

**TABLE 1:** Respondents’ age, gender and duration of practice.

Characteristics	All respondents (*n* = 420)	CHWs (*n* = 227)	CBAs (*n* = 193)	*p*
**Gender**				
Male, *n* (%)	23 (5.5)	21 (9.3)	2 (1.0)	0.000
Female, *n* (%)	397 (94.5)	206 (90.7)	191 (99.0)	
**Age (years)**				
Range	22 – 76	22 – 60	25 – 76	
Mean	45.45 ± 9.3	42.07 ± 7.6	49.41 ± 9.5	0.000
**Duration of practice (years)**				
Range	1–49	1–34	1–49	
Mean	16.12 ± 9.1	16.38 ± 8.6	16.12 ± 9.6	0.526

CHW, community health workers; CBAs, community birth attendants.

### Respondents’ knowledge of neonatal jaundice

Though all the 420 respondents had heard and seen cases of NNJ before the study visit, only 5.2% correctly defined it as ‘yellowish discolouration of the skin and other tissues of a newborn infant’. Also, only 21.9% of respondents reported that they had ever received training on NNJ management. Significantly fewer CBAs (*n* = 2; 1.0%) than CHWs (*n* = 20; 8.8%) gave accurate definitions of NNJ (*p* < 0.001). Table 2 shows the list of reported perceived signs associated with NNJ by the respondents. The most frequently reported sign of NNJ was fever; indicated by 98.4% of all respondents.

**TABLE 2:** Perceived danger signs associated with neonatal jaundice.

Characteristics	Yes/No	All participants		CHWs		CBAs		*p*
		
		*n*	%	*n*	%	*n*	%	
Fever	No	91	21.7	50	22.0	41	21.2	0.846
	Yes	329	78.3	177	78.0	152	78.8	
Refusal to feed	No	85	20.2	47	20.7	38	19.7	0.790
	Yes	335	79.8	180	79.3	155	80.3	
High pitch cry	No	205	48.8	116	51.1	89	46.1	0.300
	Yes	215	51.2	111	48.9	104	53.9	
Arching of the back	No	400	95.2	217	95.6	183	94.8	0.710
	Yes	20	4.8	10	4.4	10	5.2	
Convulsion in neonate	No	357	85.0	193	85.0	164	85.0	0.989
	Yes	63	15.0	34	15.0	29	15.0	
‘Down turning of the eye’	No	383	91.2	205	90.3	178	92.2	0.489
	Yes	37	8.8	22	9.7	15	7.8	
Severe hypotonic/hypertonia	No	396	94.3	214	94.3	182	94.3	0.990
	Yes	24	5.7	13	5.7	11	5.7	
Hard to wake/sleep	No	333	79.3	177	78.0	156	80.8	0.472
	Yes	87	20.7	50	22.0	37	19.2	

CHW, community health workers; CBAs, community birth attendants.

Table 3 shows the list of reported perceived causes of NNJ by respondents. The most frequently reported cause of NNJ by respondents was malaria in pregnancy; indicated by 81.2% of all respondents. More CBAs (22.3%) than CHWs (14.1%) mentioned that disparity in blood group is a cause of jaundice in neonates (*p* = 0.029). The list of reported treatment modalities considered effective for NNJ by respondents is given in Table 4. The most frequently reported by respondents was glucose water; indicated by 67.1% of all respondents. More CHWs (74.4%) than CBAs (57.5%) mentioned that exposure to sunlight is an effective treatment for jaundice in neonates; similarly, more CHWs (15.4%) than CBAs (5.2%) reported exchange transfusion as an effective treatment for NNJ. Table 5 shows the list of reported adverse events and morbidities related to NNJ by respondents. The most frequently reported adverse event related to NNJ by respondents was neonatal death; indicated by 96.9% of all respondents. Significantly, more CHWs (41.0%) than CBAs (23.3%) mentioned that ‘brain damage’ could be an event following poor management of jaundice in neonates (*p* < 0.001).

**TABLE 3:** Perceived causes of neonatal jaundice among respondents.

Characteristics	Yes/No	All participants		CHWs		CBAs		*p*
		
		*n*	%	*n*	%	*n*	%	
Disparity between blood groups	No	345	82.1	195	85.9	150	77.7	0.029
	Yes	75	17.9	32	14.1	43	22.3	
Sepsis	No	288	68.6	152	67.0	136	70.5	0.441
	Yes	132	31.4	75	33.0	57	29.5	
Malaria	No	79	18.8	35	15.4	44	22.8	0.054
	Yes	341	81.2	192	84.6	149	77.2	
Mosquito bite	No	362	86.2	198	87.2	164	85.0	0.505
	Yes	58	13.8	29	12.8	29	15.0	
Breast milk or germs in the breast	No	407	96.9	223	98.2	184	95.3	0.087
	Yes	13	3.1	4	1.8	9	4.7	
Prematurity	No	377	89.8	208	91.6	169	87.6	0.171
	Yes	43	10.2	19	8.4	24	12.4	
G6PD defficiency	No	397	94.5	213	93.8	184	95.3	0.500
	Yes	27	5.5	14	6.2	9	4.7	
Haemolysis in newborn	No	405	96.4	218	96.0	187	96.9	0.638
	Yes	15	3.6	9	4.0	6	3.1	

G6PD, Glucose-6-Phosphate Dehydrogenase; CHW, community health workers; CBAs, community birth attendants.

**TABLE 4:** Perceived effective treatment for neonatal jaundice stated by respondents.

Characteristics	Yes/No	All participants		CHWs		CBAs		*p*
		
		*n*	%	*n*	%	*n*	%	
Exposure to sunlight	No	140	33.3	58	25.6	82	42.5	0.000
	Yes	280	66.7	169	74.4	111	57.5	
Glucose water	No	138	32.9	74	32.6	64	33.2	0.903
	Yes	282	67.1	153	67.4	129	66.8	
Phototherapy	No	348	82.9	181	79.7	167	86.5	0.066
	Yes	72	17.1	46	20.3	26	13.5	
Exchange blood transfusion	No	375	89.3	192	84.6	183	94.8	0.001
	Yes	45	10.7	35	15.4	10	5.2	
Unripe paw-paw	No	366	87.1	201	88.5	165	85.5	0.351
	Yes	54	12.9	26	11.5	28	14.5	

CHW, community health workers; CBAs, community birth attendants.

**TABLE 5:** Adverse events and morbidities relating to neonatal jaundice stated by respondents.

Characteristics	Yes/No	All participants		CHWs		CBAs		*p*
		
		*n*	%	*n*	%	*n*	%	
Death of neonate	No	14	3.3	8	3.5	6	3.1	0.813
	Yes	406	96.9	219	96.5	187	96.9	
Brain damage	No	282	67.1	134	59.0	148	76.7	0.000
	Yes	138	32.9	93	41.0	45	23.3	
Mental retardation	No	332	79.0	175	77.1	15.7	81.3	0.288
	Yes	88	21.0	52	22.9	36	18.7	
Physical handicap	No	326	77.6	183	80.6	143	74.1	0.110
	Yes	94	22.4	44	19.4	50	25.9	
Convulsions later in life	No	358	85.2	193	85.9	163	84.5	0.677
	Yes	62	14.8	32	14.1	30	15.5	
Abnormal behaviours	No	379	90.2	202	89.0	177	91.7	0.349
	Yes	41	9.8	25	11.0	16	8.5	
Ever had about a light meter	No	407	96.9	220	96.9	187	96.9	0.988
	Yes	13	3.1	7	3.1	6	3.1	

CHW, community health workers; CBAs, community birth attendants.

Assessing the knowledge of the respondents based on their responses to questions on the definition of NNJ, colour of urine of neonates with NNJ and items listed in Tables 3, 4 and 5, a mean knowledge score of 15.5 ± 2.4 was obtained. Over three-quarters (80.2%) of respondents had poor knowledge about NNJ. Though slightly more CHWs (21.1%) than CBAs (18.1%) had good knowledge relating to NNJ, this difference was not statistically significant (*p* = 0.440). There were no significant associations between knowledge (good or poor) and each of the demographic variables—sex, religion, marital status, age, education and years of experience.

### Respondents’ practices relating to neonatal jaundice

Only 35.5% of all respondents could correctly describe the examination of a neonate for NNJ; 96.7% of all participants will check the eyes of the neonate, 71.1% said they would examine the skin for jaundice, 52.1% of respondents would check the palm or foot when examining a neonate for jaundice and 96.7% acknowledge that checking the eyes for a yellowish colour is important as part of examination for NNJ. Table 6 shows a comparison of NNJ practices with respect to examination of neonates at presentation among CHWs and CBAs. A significantly higher proportion of CHWs (24.7%) than CBAs (11.9%) reportedly practiced examination of the urine colour as an indicator for NNJ diagnosis at presentation (*p* = 0.001). However, more among CBAs than CHWs reported that they would do home follow–up after birth for neonatal illness (*p* < 0.001).

**TABLE 6:** Respondents’ responses to examination at presentation with neonatal jaundice (NNJ).

Characteristics	Yes/No	All participants		CHWs		CBAs		*p*
		
		*n*	%	*n*	%	*n*	%	
Eyes of a neonate	No	14	3.3	4	1.8	10	5.2	0.052
	Yes	406	96.7	223	98.2	183	94.8	
Looking at the skin	No	119	28.3	67	29.5	52	26.9	0.560
	Yes	301	71.7	160	70.5	141	73.1	
Looking at the palm, sole, or footsore	No	201	47.9	98	43.2	103	53.4	0.037
	Yes	219	52.1	129	56.8	90	46.6	
Colour of urine	No	341	81.2	171	75.3	170	88.1	0.001
	Yes	79	18.8	56	24.7	23	11.9	
Colour of stool	No	405	96.4	218	96.0	187	96.9	0.638
	Yes	15	3.6	9	4.0	6	3.1	
Follow up birth	No	74	7.6	55	24.2	19	9.8	0.000
	Yes	346	82.4	172	75.8	174	90.2	
Include NNJ in focused antenatal care	No	130	31.0	65	28.6	65	33.7	0.265
	Yes	290	69.0	162	71.4	128	66.3	

With respect to the treatment they would offer, a significantly higher proportion of CHWs than CBAs reportedly practice treatment of NNJ with drugs (50.2% versus 21.2%), exposure to sunlight (23.8% versus 9.8%) and glucose water (22.0% versus 8.8%); *p* = 0.000. On the other hand, fewer CHWs than CBAs would recommend local remedy (1.3% versus 8.3%) and refer to secondary health facilities (65.6% versus 86.0%); *p* = 0.000. Of the 200 (47.6%) who reported that they had treated NNJ 3 months prior to the study, 62.5% (CHWs: 66.9% and CBAs: 53.7%) treated NNJ with orthodox drugs. Approximately 58.0% (*n* = 245) of all primary healthcare workers (CHWs: 54.9%; CBAs: 64.2%) referred NNJ to secondary health facilities. The five leading prevention practices reported by respondents were malaria treatment and prevention in pregnancy (39.1%), medical screening/proper ANC/immunisation (25.7%), good personal hygiene and care in pregnancy (16.0%), good nutrition in pregnancy (10.2%) and adequate fluid intake in pregnancy (3.2%). Of the 315 respondents who reported that orthodox drugs were effective, about two-thirds mentioned antibiotics (66.9%), followed by multivitamins (20.0%), phenobarbitone (5.1%), paracetamol (4.4%) and antimalarials (3.8%). The local remedies reported included unripe paw-paw (85.7%); unripe pineapple (3.9%); cotton seed (10.4%); aloe vera (1.3%); ‘ewe ogbo, ewe ireke, ewe fruits and epo yarn’ (16.9%); mixture of Lipton tea and salt (1.3%); mixture of ampiclox and stout (1.3%); sugarcane (3.9%); bitter leaf (0.6%); prayer water (1.3%); ‘maku mixture’ (1.9%) and blue stone/lime/water (1.3%).

The overall practice score ranged from 4 to 12 with a mean practice score of 9.5 ± 1.7. Overall, slightly over half of the respondents (53.6%) engaged in the right practices and significantly more CHWs (57.3%) than CBAs (33.7%) engaged in wrong practices (*p* < 0.001). In all, significantly more CHWs (*n* = 130; 57.3%) than CBAs (*n* = 65; 33.7%) engaged in wrong practices relating to NNJ. A significantly higher proportion of female respondents (54.9%) than males (30.4%) engaged in correct practices (*p* = 0.022). Also, a significantly higher percentage of respondents aged ≤ 40 years (53.3%) than those > 40 years (43.2%) engaged in wrong practice (*p* < 0.001). Similarly, more respondents with tertiary (54.6%) than those who had secondary education or less (33.1%) engaged in wrong practice. However, there was no association between practice and religion, marital status and work experience. Of the three demographic variables (respondents’ categories, age and education) associated with practices at the levels of bivariate analyses, only respondents’ category independently predicted wrong practice (Table 7). The CHWs were 2.23 times (95% CI = 1.03, 4.80) more likely to engage in wrong practices.

**TABLE 7:** Predictors of wrong practices relating to neonatal jaundice.

Variable	UOR	95% CI of UOR	*p*	AOR	95% CI of UOR	*p*
**Workers’ category**						
CHWs	2.64	1.77, 3.92	0.000	2.23	1.03, 4.80	0.043
CBAs†	1	-	-	-	-	-
**Age**						
≤ 40	1.51	0.99, 2.27	0.051	1.18	0.76, 1.82	0.464
> 40†	1	-	-	-	-	-
**Education**						
≤ Secondary†	1	-	-	1	-	-
Tertiary education	2.43	1.61, 3.66	0.000	1.18	0.54, 2.58	0.685

† , reference category; CI, confidence interval; UOR, unadjusted odds ratio; AOR, adjusted odds ratio.

## Discussion

Three main conclusions may be made from the findings in this study. Firstly, the level of awareness about NNJ among primary health care workers in Ibadan was high, but overall assessment of level of knowledge about the disease was below average. Secondly, primary health care workers reported ominously incorrect causes of and treatments for NNJ. Thirdly, unexpected wrong practices relating to NNJ was more prevalent among CHWs than birth attendants, and the higher level of education among CHWs did not impact positively on their practice.

Though all the primary health workers who participated in this study claimed they were aware of NNJ, only 5.2% of them could accurately describe the condition. This finding corroborates findings from a previous study which revealed that primary health workers’ awareness of NNJ may not necessarily translate to good practices.^10^ However, that only a few of the respondents correctly defined NNJ is at variance to the finding of the study conducted by the same authors^10^ which showed that up to half (51.5%) of CHWs gave a correct definition of NNJ. The fact that all participants have seen at least one case of NNJ at the primary setting implies that neonates do present at primary health care level and they are often offered some form of care despite the lack of required competences and equipment to manage such cases. It is therefore important that health workers in primary health centres are able to recognise NNJ early and promptly refer to higher levels of care where correct definitive diagnosis and prompt treatments can be offered.

The overall assessment of the primary health workers’ level of knowledge of NNJ relating to cause, presentations and treatment approaches showed that approximately four out of five (80.2%) of respondents had poor knowledge. This finding suggests that most of the primary health workers were not adequately informed about NNJ. This was corroborated by the fact that only 21.9% of respondents reported that they had training on NNJ management. The finding of low knowledge among the primary health workers in the study area is at variance with an earlier report by Ogunfowora and Daniels^10^ in which the observed knowledge in many aspects of NNJ was described as ‘fairly adequate’. However, Ogunfowora and Daniels^10^ reported proportions of health workers who provided correct answers to certain question, but no aggregate scores were allotted as done in the present study. This disparity in the approach to assessment of knowledge makes it difficult to compare the findings from the two studies. The implication of poor knowledge of NNJ among primary health workers includes failure to recognise sick neonates and delay in referring such children. Also, inappropriate treatments may be given to the child and wrong counsels may be offered to mothers seeking help at that level of care. The longer term effect of such delay in diagnosis and treatment has been ascribed to as a major cause of neurodevelopmental impairment, disability and sometimes death.^16^

The data from this study suggest that knowledge was not influenced by primary health workers’ level of education. In all, the observed lack of difference in the knowledge of CHWs and CBAs raises questions on the quality of the training curriculum for CHWs and perhaps implies a need for review. Consistent with the finding in this study, previous studies also found that there was no significant association between age and knowledge about NNJ^11^ and also between level of education and knowledge about NNJ.^17^ On the contrary, the study by Sutcuoglu et al.^18^ and Egube et al.^11^ found a significant association between level of education and knowledge about NNJ while the study by Khalesi and Rakhshani^17^ also found a significant association between age and knowledge about NNJ. Similarly, a study by Balogun and Odeyemi^19^ which evaluated CBAs’ knowledge of prevention of mother-to-child transmission (PMTCT) of HIV also did not demonstrate any statistically significant association between educational status and level of knowledge.

About half (53.1%) of the respondents reported that orthodox drugs are effective in the treatment of NNJ. However, responses provided on the names of such useful drugs indicated a gross lack of knowledge on the use of medication for treatment. Whereas many respondents mentioned different drugs as being effective, only a few (7.1%) mentioned phenobarbitone. For reasons unknown, most (93.3%) respondents mentioned antibiotics as being effective; an action that amounts to abuse of drugs when the cause of NNJ is not sepsis. This finding is consistent with that of a previous study in which the authors considered it a dangerous trend as reliance on unproven medication in the management of NNJ is very hazardous.^10^ Many respondents believed in the efficacy of certain local remedies for the treatment of NNJ and a good number of them mentioned various herbal drugs as effective. There are no empirical data to justify the efficacy of local drugs in managing NNJ. Previous studies^20^ have affirmed phototherapy as the standard treatment in neonatal hyperbilirubinaemia, and exchange transfusion could be indicated for severe jaundice.

Another important finding from this study is that some myths relating to treatment modalities for NNJ exist among health workers. For instance, the primary health workers mentioned the use of ‘glucose water’, ‘exposure to sunlight’ and ‘unripe paw-paw’ for treatment of NNJ. Only a few of the respondents mentioned phototherapy and exchange blood transfusion, which are standard treatments for NNJ. Interestingly, more CHWs mentioned that ‘exposure to sunlight’ is an effective treatment of jaundice in neonates. Similarly, more CHWs than CBAs reported exchange transfusion as an effective treatment for NNJ. This is consistent with an earlier finding which revealed that 60.4% of respondents thought that ‘exposure of neonate to sunlight’ is an effective treatment.^21,22,23^ It is likely that ‘exposure to sunlight’ was likened to natural phototherapy among primary health workers in the study area.

Notably, 46.4% of the primary health workers in this study engaged in wrong practice. This finding is similar to that of an earlier study which revealed that 56% of the study respondents, had right practices.^24^ According to the standing order, primary health workers are not to treat but expected to refer cases.^21^ On the contrary, our data indicated that respondents, particularly CHWs, do engage in management of NNJ. This, however, suggests that there is higher level of adherence to guidelines (standing order) among CBAs than CHWs with respect to prompt referral of NNJ. However, it is important to note that previous studies have shown that out-born referrals to tertiary institutions had severe cases of NNJ.^15^ Similar to the finding in this study, regarding referral of NNJ cases, 22 reported that only 37.1% of health workers in primary health centres gave referrals once sick neonates presented. Nonetheless, the low referral rate among the primary health workers in the present study is at variance with a report^21^ which it is revealed that 74.2% of CHWs would immediately refer affected babies to the appropriate facilities.

As a whole, this study has generated data that provide insight into what is currently being done, why and how newborns with NNJ are being managed by CHWs and CBAs. With these data, interventions that would enhance the knowledge, attitude and practice of health workers regarding NNJ in the light of the local circumstances can be proffered. Furthermore, these data provide useful background for planning training activities that better suit the CHWs and CBAs practicing in Ibadan, Nigeria. Accessible literature suggests that this study is the first to be conducted on the knowledge and practices relating to NNJ among CHWs and CBAs in Ibadan, Nigeria. Also, the fact that an attempt was made recruit all eligible primary health workers on duty during the study period lends credence to the generalisability of our findings. In addition, the number of participants in the study is far above the number recruited in a similar study by Ogunfowora and Daniels.^10^ The present study also included CBAs and assessed knowledge of primary health workers by adapting a constructed scale which no other study from Nigeria has reported.

However, a few factors may limit the generalisability of our findings. Firstly, the extent to which health workers responded accurately to the items in the questionnaire and whether their responses truly reflect the actual level of knowledge and practices relating to NNJ could not be verified. Nonetheless, the findings are correct within the limit of the validity and reliability of the questionnaire. It is important to note that the questionnaire was designed with items drawn from a previous study and pretested for validity in a population relatively similar to the study population. In addition, the questionnaire used in this study was translated into the local language (Yoruba) for the purpose of enhancing the understanding of respondents. These should expectedly improve the quality of the data. Secondly, the similarities or otherwise of the health workers who did not participate in the study remains unknown. The researchers made effort to capture all primary health workers at their duty post during the study period.

## Conclusions

The findings of this study suggest that there is a need to intensify efforts at improving the knowledge of NNJ among primary health workers in Ibadan through in-service training on a scheduled basis in order to address specific needs such as referral and correct treatments for NNJ. This training should be all-inclusive and should involve the CBAs using teaching and learning methods that are tailored to their level of education. Also, the LGAs health department should scale up efforts at monitoring the activities of CBAs to protect the lives of their clients who invariably ‘must’ patronise them. Synergy between the birth homes and the primary health centres is also vital to reducing the impact of NNJ on child health and well-being.
